# Knockdown of BAP31 Suppresses Tumorigenesis and Stemness in Breast Cancer Cells via the Hippo Pathway

**DOI:** 10.3390/ijms26083576

**Published:** 2025-04-10

**Authors:** Zhenzhen Hao, Bo Zhao, Fei An, Wanting Zhang, Xiaoshuang Zhu, Shihao Meng, Bing Wang

**Affiliations:** Institute of Biochemistry and Molecular Biology, College of Life and Health Sciences, Northeastern University, Shenyang 110819, China; 1710065@stu.neu.edu.cn (Z.H.); 1810068@stu.neu.edu.cn (B.Z.); 2201435@stu.neu.edu.cn (F.A.); 2110482@stu.neu.edu.cn (W.Z.); 2010494@stu.neu.edu.cn (X.Z.); 2310514@stu.neu.edu.cn (S.M.)

**Keywords:** BAP31, MST1, tumorigenesis, stemness, PCMT1

## Abstract

The enhancement of stemness in cancer cells is correlated with the malignancy level in human cancers. B cell receptor-associated protein 31 (BAP31) has been implicated in tumor progression; however, its specific role in breast cancer remains unclear. This study aimed to elucidate the biological function and molecular mechanisms of BAP31 in tumorigenesis and cancer stemness. Cancer stemness was assessed through tumor sphere formation and flow cytometry assays. Western blot analysis was employed to examine alterations in core stemness factors in BAP31 knockdown cell lines, in order to explore potential underlying mechanisms. Finally, we explored the role of BAP31 by developing xenograft models using nude mice in vivo. Our findings revealed that BAP31 expression was elevated in breast cancer cells, and its knockdown led to a decrease in both sphere formation and the CD44+CD24− population. Furthermore, the knockdown of BAP31 significantly diminished the expression of core stemness factors, such as Sox2 and c-Myc, in breast cancer cells in vitro. Consistently, the suppression of BAP31 markedly inhibited the tumorigenicity and stemness of breast cancer in vivo. The functional analysis further indicated that the knockdown of BAP31 diminishes stemness by activating the Hippo pathway kinase MST1 and inhibiting the transcription factor YAP. Notably, our study was the first to demonstrate that BAP31 interacts with PCMT1, a direct negative regulator of MST1 kinase. These findings identify BAP31 as a regulator of the Hippo pathway, highlighting its critical role in breast cancer tumorigenesis and stemness. Consequently, BAP31 emerges as a potential therapeutic target for this malignancy.

## 1. Introduction

Breast cancer represents one of the most prevalent malignancies among women globally, with its aggressiveness closely associated with the stemness of cancer cells. Cancer cell stemness, often referred to as cancer stemness, denotes the capacity of tumor cells to self-renew and differentiate, thereby significantly contributing to tumor initiation, progression, metastasis, and resistance to therapeutic interventions [1]. Research has indicated that breast cancer stem cells (BCSCs) constitute a minor population of cells exhibiting stem cell-like properties, which are capable of initiating tumor formation and recurrence [2]. The presence of breast cancer stem cells (BCSCs) is intricately associated with tumor invasion, metastasis, and resistance to chemotherapy. BCSCs are characterized by an elevated expression of stem cell markers, including CD44+CD24−, as well as a notable resistance to chemotherapeutic agents [3]. These cells possess the ability to form spheroids (mammospheres) in vitro and can also generate tumors in vivo, thereby underscoring their robust tumorigenic potential [4]. Consequently, elucidating the mechanisms underlying the formation of BCSCs is of paramount importance.

B-cell receptor-associated protein 31, a 28 kDa endoplasmic reticulum chaperone protein, is evolutionarily conserved and widely expressed. Identified by Kim et al. in 1994, BAP31 is a member of the B cell receptor family of proteins [5]. The intact BAP31 protein molecule is involved in B cell activation, membrane protein trafficking, and apoptosis regulation [6,7]. Furthermore, BAP31 plays crucial roles in mitochondrial homeostasis, autophagy, immune system function, and nervous system regulation [8,9,10,11]. Currently, a growing body of evidence indicates a strong association between BAP31 and cancer progression. For instance, in cervical cancer, inhibiting BAP31 has been shown to impede the advancement and spread of the disease [12]. The downregulation of BAP31 has been found to decrease SERPINE2 expression and hinder the growth and proliferation of hepatocellular carcinoma in animal models [13]. Our previous research has demonstrated that BAP31-mediated miR-206/133b Cluster facilitates the transendothelial migration and metastasis of colorectal cancer [14]. Moreover, disrupting the interaction between BAP31 and p27 using an intracellular antibody has been shown to induce cell cycle arrest and promote cell death in gastric cancer cells [15]. However, the roles and related mechanisms of BAP31 in regulating the stemness and tumorigenesis remain unclear in breast cancer.

In the context of breast cancer treatment, strategies aimed at targeting breast cancer stem cells (BCSCs) are increasingly garnering attention. Research indicates that specific cell signaling pathways, including the Wnt, Notch, and Hedgehog pathways, play a crucial role in the self-renewal and differentiation processes of BCSCs [16]. Targeting these pathways could enhance the therapeutic efficacy of breast cancer treatments and potentially decrease the risk of recurrence and metastasis [17]. The Hippo pathway, an evolutionarily conserved signaling cascade, plays a crucial role in cancer development and organ growth. Recent research suggests that the Hippo pathway also contributes to tumorigenesis and the self-renewal of cancer stem cells [18,19]. This pathway includes two core kinases (MST1/2 and LATS1/2), YAP/TAZ, and other adapter ligands, and is activated by upstream signals that lead to the phosphorylation of MST1/2 and the activation of LATS1/2, ultimately inhibiting YAP activity [20]. Studies have demonstrated that the transcriptional co-activators YAP and TAZ can enhance the expression of genes associated with stemness and tumorigenesis when the Hippo pathway is inhibited [21]. MST1, a serine-threonine kinase encoded by the STK4 gene, is predominantly located in the cytoplasm and plays a crucial role in activating the downstream signaling pathway of Hippo. The autophosphorylation of Mst1 is believed to contribute to its kinase activation, while protein interactions also play a significant role in regulating the Mst1 activity. Various proteins, such as hWW45 [22,23], PHLPP1 [24], and Hsp70 [25], have been identified as interacting with Mst1 and influencing its activation. The gene PCMT1, responsible for encoding the protein repair enzyme protein-L-isoaspartate O-methyltransferase, serves as a direct negative regulator of the MST1 kinase [26].

In this study, we demonstrated that the knockdown of BAP31 leads to the downregulation of PCMT1, thereby modulating MST1 activation and the subsequent Hippo/YAP signaling pathway. Furthermore, we provide the novel evidence that BAP31 interacts with PCMT1, initiating a cascade of effects on breast cancer cells. Therefore, BAP31 might serve as a therapeutic target to attenuate the tumorigenesis and eliminate the stemness of breast cancer cells.

## 2. Results

### 2.1. High Expression of BAP31 Associated with Poor Prognosis in Breast Cancer

**Figure 1:**
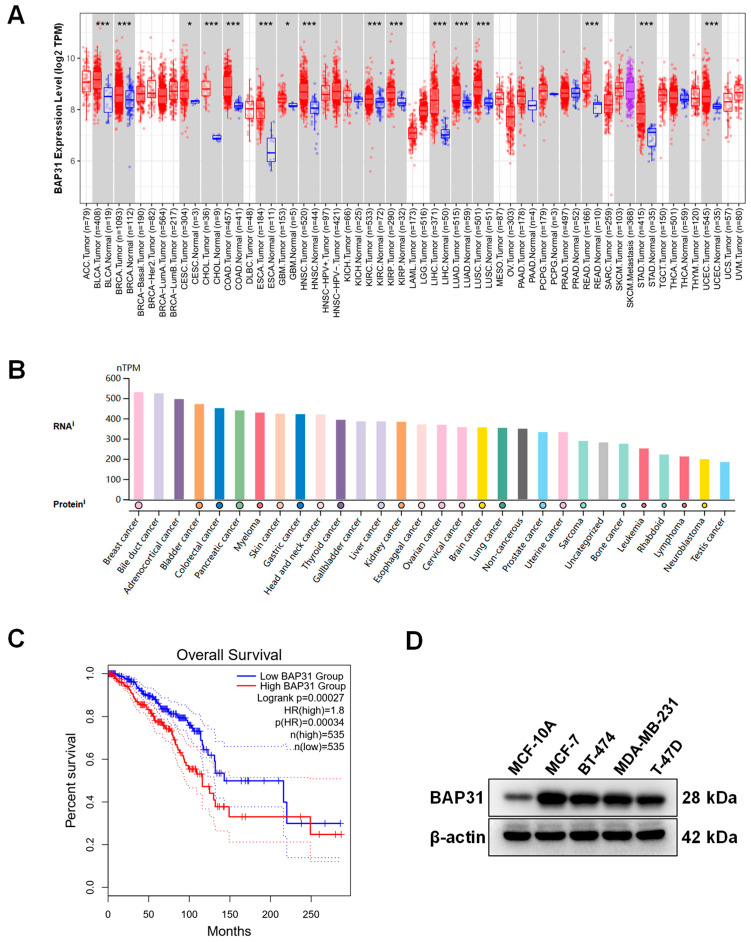
High BAP31 expression was significantly associated with poor prognosis. (**A**) The differential expression of BAP31 between tumor and normal tissues in pan-cancer analysis. (**B**) Expression of BAP31 in various cancer cell lines. (**C**) Kaplan–Meier survival analysis of BAP31 expression in breast cancer. (**D**) Western blot was used to analyze BAP31 in MCF-10A, MCF7, BT-474, MDA-MB-231, and T-47D cells (n = 3). * *p* < 0.05; *** *p* < 0.001.

Initially, to investigate the expression level of BAP31, we conducted an analysis using the TIMER 2.0 database on the TCGA datasets. The results suggested that BAP31 was significantly upregulated in BRCA compared with in adjacent normal tissues (*p* < 0.001) (Figure 1A). It was further found that BAP31 was upregulated in most cancer cells, with highest expression in breast cancer cell lines (Figure 1B). In addition, Kaplan–Meier analysis revealed that the elevated BAP31 expression was associated with poorer prognosis in breast cancer (Figure 1C). Moreover, Western blot analyze indicated that the expression of BAP31 in breast cancer cell lines including MCF-7, BT-474, MDA-MB-231, and T-47D was higher than that in normal breast epithelial cells (MCF-10A) (Figure 1D). These findings showed that the BAP31 expression was significantly upregulated in breast cancer and its expression level was associated with poor prognosis.

### 2.2. BAP31 Plays an Important Role in Breast Cancer Tumorigenesis and Stemness

**Figure 2:**
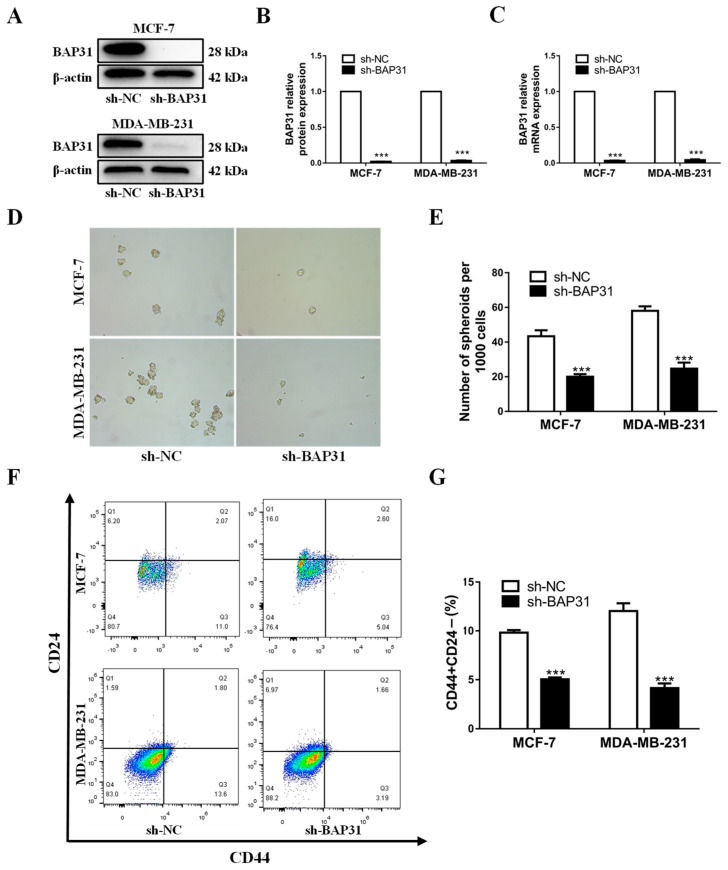
BAP31 has an important role in breast cancer cells tumor sphere formation and stemness. (**A**) Western blot was used to analyze BAP31 in MCF-7 and MDA-MB-231 cells. (**B**) Relative content analysis of BAP31 proteins in MCF-7 and MDA-MB-231 cells. (**C**) Real-time PCR was used to analyze the expression of BAP31 in MCF-7 and MDA-MB-231 cells. (**D**,**E**) Representative images of sphere formation assay of MCF-7 and MDA-MB-231 cells. (**F**,**G**) CD44+CD24− cell population of MCF-7 and MDA-MB-231 cells was analyzed by Flow Cytometry. N = 3. *** *p* < 0.001.

BAP31 has been demonstrated to facilitate cancer progression in various human malignancies, such as ovarian, cervical, and colorectal carcinoma. However, the potential involvement of BAP31 in breast cancer progression remains uncertain. Cancer stemness represents one of the most pivotal potential mechanisms underpinning the tumorigenesis and progression of breast cancer. This study utilized the cell lines MCF7 and MDA-MB-231 to investigate the effects of BAP31 knockdown using lentiviral vectors encoding shRNA. Analysis through Western blot and quantitative real-time PCR indicated a significant depletion of BAP31 in both cell lines (Figure 2A–C). Subsequent breast cancer sphere formation assays demonstrated a notable decrease in the number of breast cancer spheres in MCF7 and MDA-MB-231 cells lacking BAP31 (Figure 2D,E). In addition, flow cytometric analysis revealed a reduction in the proportion of CD44+CD24− cells subsequent to BAP31 knockdown (Figure 2F,G). These findings suggest that BAP31 may play a role in the regulation of tumorigenesis and stemness in breast cancer cells.

### 2.3. The Relationship of BAP31 with Core Stemness Factors

**Figure 3:**
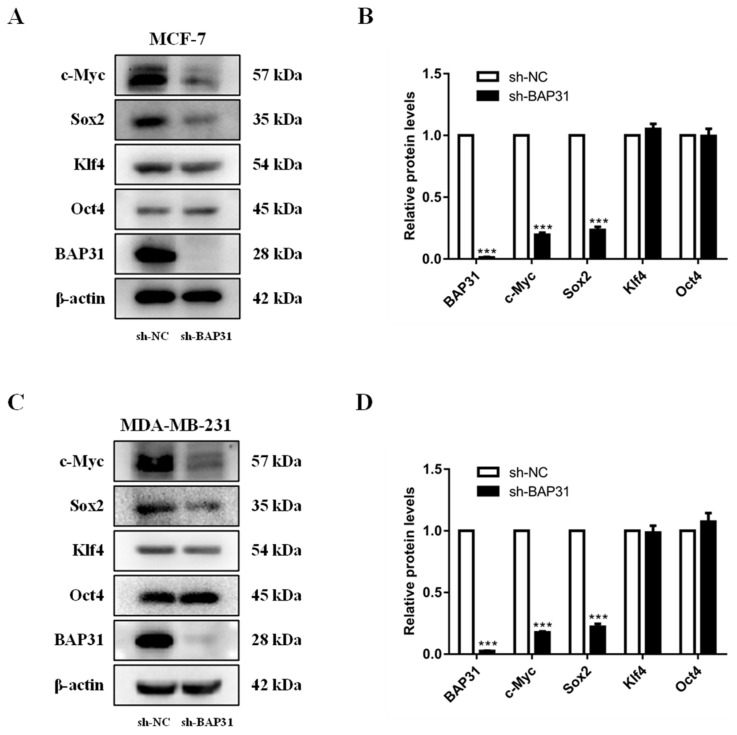
The relationship of BAP31 with core stemness factors. (**A**,**B**) Western blot analysis of BAP31, c-Myc, Sox2, Klf4, and Oct4 were performed on the lysates of sh-NC, sh-BAP31 in MCF-7 cells. (**C**,**D**) Western blot analysis of BAP31, c-Myc, Sox2, Klf4, and Oct4 were performed on the lysates of sh-NC, sh-BAP31 in MDA-MB-231 cells. N = 3. *** *p* < 0.001.

We further analyzed the relationship between BAP31 and stemness in breast cancer cells. Sox2, c-Myc, Klf4, and Oct4 are four core stemness factors that play pivotal roles in maintaining cancer cell stemness. These factors are not only essential for inducing pluripotent stem cells (iPSCs) but are also critically involved in cancer development and progression. Western blot analysis demonstrated a reduction in the expression of core stemness factors Sox2 and c-Myc upon BAP31 knockdown in MCF7 and MDA-MB-231 cells (Figure 3A–D). And there were no significant changes in the expression of Klf4 and Oct4 protein in BAP31 knockdown cells (Figure 3A–D). In summary, the decreased stemness caused by BAP31 knockdown may be closely related to Sox2 and c-Myc.

### 2.4. Knockdown of BAP31 Suppresses Tumorigenesis and Stemness of Breast Cancer Cells In Vivo

**Figure 4:**
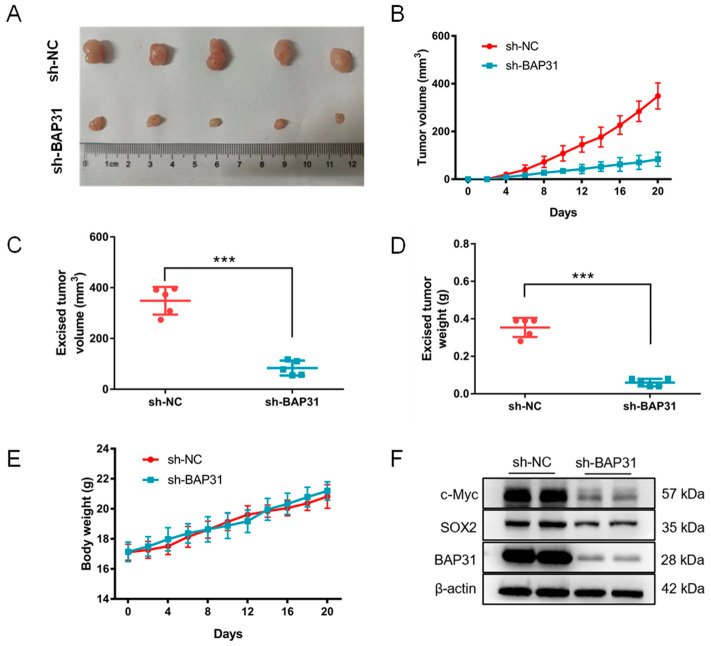
Knockdown of BAP31 suppresses tumorigenesis and stemness of breast cancer cells in vivo. (**A**) Digital images of tumor tissues from different experimental groups. (**B**) The change curve of tumor volume. (**C**) The average tumor volume was measured after the mice were sacrificed. (**D**) The measurement of tumor weight. (**E**) The variation of body weight in each group. (**F**) Western blot analysis of BAP31, c-Myc, and Sox2 proteins in tumor tissues. β-actin was used as loading control. N = 5. *** *p* < 0.001.

To assess the functionality of BAP31 in vivo, we established stable BAP31 knockdown MDA-MB-231 cells, which were subsequently implanted subcutaneously into BALB/c nude mice. Tumor volume and weight were then quantified in the xenograft mouse models. Our results, depicted in Figure 4A,B, demonstrate that the knockdown of BAP31 effectively inhibited tumor growth and size compared to the control group (sh-NC). Following the excision of tumor tissues, calculations of tumor volumes and weights revealed a significant decrease in both parameters in the sh-BAP31 group compared to the sh-NC group (Figure 4C,D). As illustrated in Figure 4E, the fluctuations in body weight in the sh-BAP31 group were comparable to those observed in the control group. Consistent with the findings of the in vitro cell-based study, the attenuation of stemness induced by BAP31 knockdown was evident in tumor tissues. The protein expression levels of SOX2 and c-Myc were significantly reduced in the sh-BAP31 group compared to the sh-NC group (Figure 4F). These results indicate that the suppression of BAP31 inhibits breast cancer tumorigenesis and stemness in an in vivo setting.

### 2.5. BAP31 Regulates the Hippo Pathway

**Figure 5:**
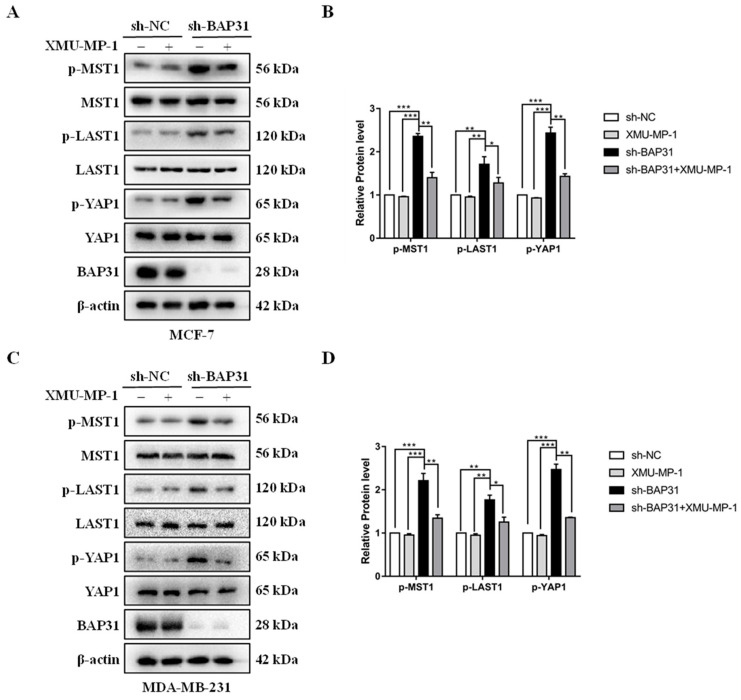
BAP31 regulates the hippo pathway. (**A**) Western blot showed the protein expression levels of MST1, p-MST1, LAST1, p-LAST1, YAP1, and p-YAP1 in MCF-7 cells. (**B**) Relative quantification analysis of p-MST1, p-LAST1, and p-YAP1 in MCF7 cells. (**C**) Western blot showed the protein expression levels of MST1, p-MST1, LAST1, p-LAST1, YAP1 in MDA-MB-231 cells. (**D**) Relative quantification analysis of p-MST1, p-LAST1, and p-YAP1 in MDA-MB-231 cells. N = 3. * *p* < 0.05, ** *p* < 0.01, *** *p* < 0.001.

Sox2 and c-Myc have been identified as the downstream target genes of the Hippo signaling pathway, which has been linked to tumor progression when dysregulated. Once this pathway is activated, the upstream kinase phosphorylates YAP, inhibiting its activity and downstream gene expression. In order to investigate the potential regulation of the Hippo pathway by BAP31, Western blot analysis was conducted to assess the levels of key proteins involved in the pathway, including MST1, p-MST1, LAST1, p-LAST1, YAP1, and p-YAP1, in MCF7 and MDA-MB-231 cells transfected with sh-BAP31 and sh-NC. As a consequence of BAP31 knockdown, the protein levels of p-Mst1, p-Lats1, and p-YAP1 were notably elevated, while the total protein levels of MST1, LAST1, and YAP1 remained unchanged, as shown in Figure 5A–D. When we used the specific MST1 phosphorylation inhibitor XMU-MP-1, the phosphorylation level of sh-BAP31 group was significantly reduced in Figure 5A–D. These results indicate that BAP31 may function in breast cancer by modulating the Hippo pathway.

### 2.6. BAP31 Interacts with PCMT1 and Regulates Its Expression

**Figure 6:**
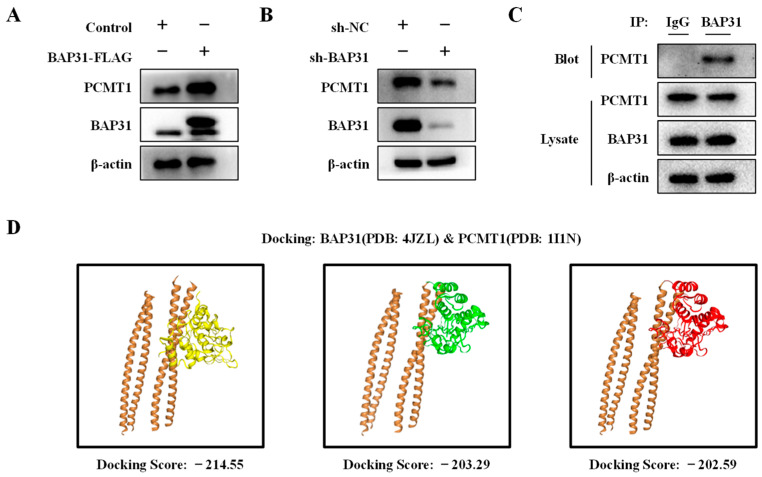
BAP31 positively regulates PCMT1 and interacts with PCMT1. (**A**) The overexpression of BAP31 increased the expression of PCMT1. (**B**) Knockdown of BAP31 decreased the expression of PCMT1. (**C**) MCF7 cells were lysed in CHAPS buffer, and immunoprecipitation (IP) was performed with an anti-BAP31 antibody. The association between BAP31 and PCMT1 was monitored by immunoblotting with an anti-PCMT1 antibody. (**D**) Predicted models of BAP31-PCMT1 protein docking. The top three homologous docking models for BAP31 and PCMT1 interaction were predicted by HDOCK server online and presented with three differentially colored 3D structures of PCMT1. N = 3.

Previous studies have shown that the interaction between PCMT1 and MST1 negatively regulates MST1 activity [26]. The current study investigates the potential role of BAP31 in regulating breast cancer progression through its interaction with PCMT1. To assess the relationship between BAP31 and PCMT1, we detected the expression levels of BAP31 in MCF7 cells through overexpression and knockdown experiments. Our results demonstrated that the overexpression of BAP31 leads to an increase in PCMT1 protein levels, while the knockdown of BAP31 results in a decrease in PCMT1 protein levels (Figure 6A,B). Subsequently, we conducted an immunoprecipitation assay using MCF7 cells to further investigate this interaction. Pooled fractions containing both BAP31 and PCMT1 were subjected to immunoprecipitation using the anti-BAP31 antibody, followed by the examination of the specific immunocomplexes via immunoblotting with PCMT1. A band corresponding to the expected size of PCMT1 was clearly detected on the blot when hydrophilic surfactant (CHAPS) was employed, indicating the potential interaction between BAP31 and PCMT1 (Figure 6C). HDOCK Server analysis found that there were many pairs of interactions between BAP31 and PCMT1 proteins, and the binding regions of the top three scores are shown in Figure 6D. This suggests that BAP31 and PCMT1 may exert biological functions through their interactions in breast cancer.

### 2.7. Restoration of BAP31 Protein Increased the Expression of PCMT1 and Decreased p-MST1 Protein

**Figure 7:**
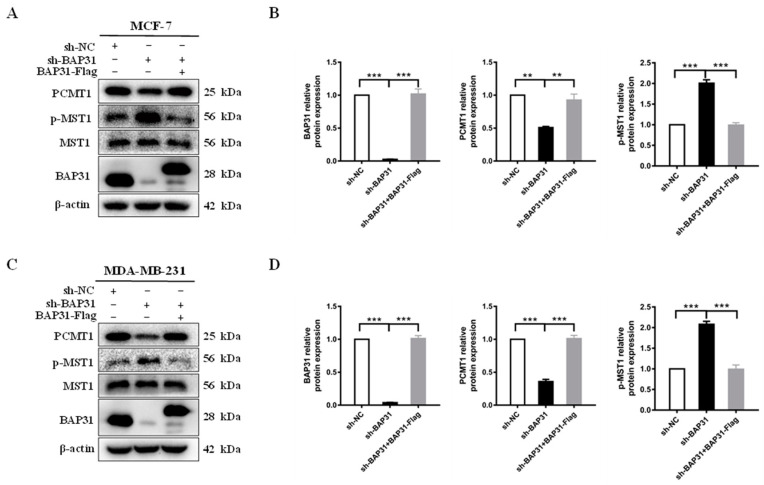
Restoration of BAP31 protein increases the expression of PCMT1 and decreases p-MST1 protein. (**A**,**C**) Western blot analysis of BAP31, PCMT1, and p-MST1 was performed in MCF-7 and MDA-MB-231 cells, respectively. (**B**,**D**) Relative content analysis of BAP31, PCMT1, and p-MST1 proteins in MCF-7 and MDA-MB-231 cells, respectively. N = 3. ** *p* < 0.01, *** *p* < 0.001.

To further investigate the impact of BAP31 on the protein expression of PCMT1 and p-MST1, a BAP31-Flag plasmid was transfected into sh-BAP31 breast cancer cells in logarithmic growth phase, followed by Western blot analysis to assess the protein levels of PCMT1 and p-MST1. The results depicted in Figure 7A–D indicate a significant decrease in PCMT1 protein levels and a notable increase in p-MST1 protein levels in the sh-BAP31 group compared to the control group. However, the restoration of BAP31 in the sh-BAP31 group led to a significant increase in the protein expression of PCMT1 and a significant decrease in the protein expression of p-MST1. These findings suggest that BAP31 plays a role in regulating the protein levels of PCMT1 and p-MST1.

## 3. Discussion

Despite the recent advancements in therapy aimed at extending survival rates, breast cancer continues to be the most prevalent malignancy posing a significant threat to women’s health [27]. The enhanced comprehension of the pathogenesis of breast cancer could facilitate the identification of novel therapeutic targets for improved treatment outcomes. BAP31 has been found to be overexpressed in various cancer types, including breast cancer [28], yet its specific role and the underlying mechanisms in breast cancer remain unclear. The significance of our current study lies in its pioneering demonstration of BAP31’s involvement in promoting breast cancer tumorigenesis and stem cell proliferation, as well as in establishing a direct association between the BAP31 and Hippo pathway. Our findings suggest that BAP31 may represent a crucial target for therapeutic intervention in breast cancer.

BAP31 plays a crucial role in regulating various biological processes within tumors, as evidenced by recent research highlighting its significant impact on cancer progression [28,29,30]. The emerging recognition of BAP31 as a promising therapeutic target for cancer is supported by studies demonstrating its promotion of proliferation, invasion, and metastasis in liver cancer cells [13], as well as its ability to induce cell death in gastric cancer through the regulation of p27 proteasome degradation [15]. Additionally, BAP31 has been implicated in neuroblastoma angiogenesis by modulating the GAL-3 and VEGF signaling pathways [31]. Breast cancer is a malignant tumor and a leading cause of cancer-related morbidity and mortality among women worldwide. Our findings revealed a high expression of BAP31 in breast cancer cells, prompting an investigation into its role in regulating tumorigenicity and stemness. The depletion of BAP31 resulted in a significant reduction in sphere formation and stemness of breast cancer cells, indicating a potential association between BAP31 and the tumorigenicity and stemness of these cells.

Cancer stem cells (CSCs) exhibit traits of self-renewal and multipotent differentiation, potentially contributing to tumorigenesis, resistance to treatment, recurrence, and metastasis [32]. These cells employ various self-defense mechanisms to withstand therapeutic interventions. Fundamentally, CSCs represent the foundational “roots” of aggressive tumors, for which effective treatments remain elusive [33]. Emerging research underscores the role of CSCs in breast cancer as facilitators of tumor expansion, resistance to therapy, and disease advancement [34,35,36]. Our study reveals that the knockdown of BAP31 effectively inhibits tumor growth and stemness in both in vitro and in vivo settings, suggesting that targeting BAP31 may serve as a promising therapeutic approach for the treatment of breast cancer.

The biological activity of cancer stem cells (CSCs) is governed by essential stemness factors, such as Sox2 and c-Myc, which are pivotal for preserving stem cell characteristics and facilitating cellular proliferation. Yes-associated protein (YAP) serves as a critical effector within the Hippo signaling pathway, primarily governing a range of biological processes, including cell proliferation, survival, and tissue growth. YAP is also instrumental in modulating stem cell characteristics and preserving cell fate, particularly through its regulation of key transcription factors such as Sox2 and c-Myc [37]. Sox2 is a pivotal transcription factor essential for maintaining the self-renewal and pluripotency of stem cells. Research has demonstrated that YAP enhances the transcriptional activity of Sox2 through its interaction with TEAD transcription factors, consequently augmenting the cell stemness [38]. In the context of tumor biology, the dysregulated activation of YAP is significantly associated with the initiation and progression of various cancers. YAP facilitates tumor cell proliferation and metastasis by upregulating oncogenes such as c-Myc [39]. Therefore, YAP is pivotal not only in normal physiological processes but also in tumorigenesis [40,41]. In summary, YAP, serving as a central effector protein within the Hippo signaling pathway, influences cell proliferation and fate determination by modulating the expression of Sox2 and c-Myc, thereby playing a significant role in both development and tumorigenesis. Our study revealed that the knockdown of BAP31 led to a decrease in the expression of Sox2 and c-Myc, thereby establishing a connection between BAP31 and cancer stemness. Nevertheless, the regulatory mechanism through which BAP31 influences the Hippo/YAP pathway remains insufficiently elucidated and requires further investigation.

The Hippo pathway has been demonstrated to play a role in tumorigenesis and stemness in various cancers, including lung [42], gliomas [43], melanoma [44], and liver cancers [45]. Upon the activation of the Hippo pathway, MST1/2 is phosphorylated and activated, leading to the phosphorylation of its substrate LATS1/2. This in turn phosphorylates the downstream effector YAP, resulting in the inhibition of YAP activity and the expression of downstream targets [46]. In the current study, we observed that knockdown of BAP31 led to the inhibition of YAP downstream target genes (Sox2 and c-Myc) in both BAP31 knockdown cells and in vivo xenograft models of knockdown cell lines. Subsequently, the expression levels of genes upstream were assessed, revealing that the knockdown of BAP31 resulted in the increased phosphorylation of MST1, LATS1, and YAP1, while the total protein levels of MST1, LATS1, and YAP remained unchanged. These findings indicate a favorable influence of BAP31 on the Hippo pathway.

Protein interactions have been demonstrated to be crucial in modulating the activity of Mst1. PCMT1 has been identified as an interacting protein of MST1 and is shown to directly inhibit pmst1 [26]. Our research indicates a positive correlation between BAP31 and PCMT1, with evidence of mutual interaction. This investigation underscores the role of BAP31 as a critical regulator of the Hippo pathway, as it interacts with PCMT1, which directly associates with the kinase domain of MST1. This interaction results in the inhibition of MST/LATS kinase activity and the activation of the oncogenic cofactor YAP. Additionally, the depletion of BAP31 significantly impedes breast cancer tumorigenesis and stem cell growth, highlighting the importance of BAP31 as a crucial therapeutic target for breast cancer. However, the impact of BAP31 on PCMT1 needs to be studied further.

In summary, our research has elucidated a mechanism by which BAP31 modulates tumorigenesis and stemness through the Hippo pathway in breast cancer cells. Specifically, we have found that BAP31 interacts with PCMT1, and depletion of BAP31 leads to the inhibition of PCMT1, consequently promoting MST1 kinase activity and ultimately enhancing the Hippo pathway. These findings suggest that targeting BAP31 may hold promise as a therapeutic strategy for the treatment of breast cancer.

## 4. Materials and Methods

### 4.1. Analysis of the Expression Level of BAP31 in the Pan-Cancer Datasets and Prognosis Analysis in Breast Cancer

The expression levels of the BAP31 gene in a range of cancer tissues were acquired using the “Gene_DE” module available in TIMER 2.0 (http://timer.cistrome.org/, accessed on 15 November 2024). The Human Protein Atlas (HPA; www.proteinatlas.org, accessed on 21 November 2024) was utilized to investigate the expression of the BAP31 in most cancer cells. The Kaplan–Meier Plotter (https://kmplot.com/, accessed on 23 November 2024) was utilized to examine breast cancer prognosis.

### 4.2. Cell Culture and Chemicals

The human breast cancer cell lines MCF7, MDA-MB-231, BT-474, and T-47D were obtained from the cell bank of the Chinese Academy of Sciences (Shanghai, China). MCF7 and MDA-MB-231 were cultured in MEM medium (Gibco, Grand Island, NY, USA) and L-15 medium (Gibco), respectively, BT-474 and T-47D were maintained in RPMI1640 medium (Gibco). All mediums were supplemented with 10% fetal bovine serum, 1% l-glutamine, and 1% penicillin–streptomycin (Beyotime, Shanghai, China). The cells were maintained at 37 °C in a CO_2_ incubator (Thermo Fisher Scientific, Waltham, MA, USA). XMU-MP-1 was purchased from MedChemExpress (MCE, Monmouth Junction, NJ, USA).

### 4.3. Construction of Stable Cell Lines

Cells were rendered with a stable knockdown of BAP31 through lentivirus infection, as outlined in a previous study [15]. Specifically, HEK-293T cells were co-transfected with pLKO.1-puro-shBAP31 and packaging vectors (psPAX2, pMD2.G) using Lipo8000 transfection reagent (Beyotime, Shanghai, China). Following a 48 h infection period, the cells underwent selection in a puromycin-containing medium for a duration of 1 month. All transfection protocols adhered to the manufacturer’s guidelines.

### 4.4. Western Blot Analysis

Proteins were extracted from cells using RIPA lysis buffer (Beyotime, Shanghai, China) supplemented with protease inhibitor cocktail and phosphatase inhibitors NaF and Na3VO4. The obtained lysates were then subjected to separation via SDS-PAGE and transferred onto PVDF membranes (Millipore, Burlington, MA, USA). The membrane was blocked with 5% non-fat milk and subsequently incubated with primary and secondary antibodies in sequence. The immunoreactive bands were observed using Bio-Rad ChemiDoc™ Imaging Systems with an ECL detection kit (Thermo Fisher Scientific, Waltham, MA, USA). The Image Lab software (5.2.1, Bio-Rad, Hercules, CA, USA) was utilized for quantification.

### 4.5. Quantitative Real-Time PCR

Total RNA was extracted using TRIzol from Tiangen Biotech (Beijing, China), followed by cDNA synthesis with a Kangwei reverse transcription kit. Quantitative RT-PCR was performed using Kangwei 2× SYBR Green qPCR Master Mix on a CFX96 system, with the results expressed as fold change relative to GAPDH (2^−ΔΔCt^). PCR primers were obtained from Shanghai Sangon (Shanghai, China), with sequences for BAP31 as F-CCTCTATGCGGAGGTCTTTGT and R-CCGTCACATCATCATACTTCCGA, and for GAPDH as F-GACAGTCAGCCGCATCTTCT and R-TTAAAAGCAGCCCTGGTGAC.

### 4.6. Tumorsphere Formation Assay

The generation of spheres was conducted in ultralow attachment six-well plates (Corning, Corning, NY, USA) supplemented with 1 × B27, 20 ng/mL bFGF, and 20 ng/mL EGF. Cells were seeded at a density of 1 × 10^4^ cells/well and maintained at 37 °C with 5% CO_2_. Following a 10-day incubation period, the diameters of each sphere were assessed, with those exceeding 100 um considered primary spheres. The subsequent passaging of primary spheres to secondary spheres was achieved using trypsin 0.5% and EDTA, followed by plating the cells into 24-well ultralow attachment culture plates at a density of 10^3^ per well. Additionally, cells were cultured in stem cell media and incubated for an additional 10 days to generate secondary spheres.

### 4.7. Flow Cytometry Analysis

The breast cancer stem cell population was characterized utilizing flow cytometry to identify cells expressing CD44 and CD24 markers. A total of 1 × 10^6^ cells were harvested and resuspended in 100 μL PBS, followed by labeling with APC-conjugated CD44 antibody and FITC-conjugated CD24 antibody. Subsequently, the reaction mixture was incubated at room temperature in the dark for 30 min. Analysis was performed using the BD LSR II Flow Cytometer (BD Biosciences, Franklin Lakes, NJ, USA), with cancer cells exhibiting a high CD44 and a low CD24 expression being identified as the stem cell population.

### 4.8. Immunoprecipitation (IP) Assay

Breast cancer cells were subjected to lysis using IP buffer for a duration of 30 min in an ice bath. The resulting lysates were then centrifuged to isolate the supernatants, which were subsequently incubated with antibodies overnight at a temperature of 4 °C. Following this, the mixture was exposed to protein G agarose beads (Invitrogen, Carlsbad, CA, USA) for a period of 2 h. Upon the addition of protein G agarose beads, the complexes underwent centrifugation, were washed five times, resuspended in SDS loading buffer, and subjected to boiling for 10 min. The resulting supernatants were then loaded onto an SDS-PAGE gel, followed by electrophoresis and analysis using Western blotting techniques, as outlined previously.

### 4.9. Protein–Protein Docking

The PDB format file of the docking protein structure was downloaded from the RCSB PDB database for storage. HDOCK was used for protein–protein docking. Login the HDOCK online docking tool (http://hdock.phys.hust.edu.cn/ accessed on 26 December 2024). Open the PDB format file of the receptor protein BAP31 in the Input Receptor Molecule project. Select the PDB format file of the ligand protein PCMT1 in the Input Ligand Molecule project to open. Click Submit. After the calculation is completed, the 3D pattern map of receptor ligand binding is recorded, and the docking score is recorded. The smaller the score, the higher the affinity.

### 4.10. Xenograft Tumors in Nude Mice

In this investigation, female BALB/c nude mice aged 5 weeks were procured from SPF Biotechnology Co., Ltd. (Beijing, China) and were maintained in a controlled environment free of specific pathogens. MDA-MB-231 cells, either with or without BAP31 knockdown, were combined with Matrigel matrix (1:1, BD Biosciences, Franklin Lakes, NJ, USA) at a concentration of 5 × 10^6^ cells and subsequently subcutaneously injected into the dorsal region of the nude mice. Concurrently, the progression of tumor volume and body weight in the nude mice was monitored and documented. Tumor volumes were calculated using the formula: V (mm^3^) = (length × width^2^)/2. At the conclusion of the experimental period, tumor tissues were harvested, weighed, and collected. No accidental deaths of mice occurred during the experiment, and the mice exhibited healthy vital signs throughout.

### 4.11. Statistical Analysis

All experimental data were analyzed utilizing SPSS version 17.0 statistical software. A student’s *t*-test was employed to compare the means between two groups of independent samples. The data are presented as the mean ± standard deviation (SD) from at least three independent experiments. All histograms were created using GraphPad Prism 7.0 (San Diego, CA, USA). A *p*-value of less than 0.05 was considered indicative of statistical significance.

## Data Availability

The data that support the findings of this study are available from the corresponding author upon reasonable request.
